# 

**Published:** 1898-03-19

**Authors:** 


CADBURY'S COCOA ABSOLUTELY PURE.

CADBURY'S COCOA NO DRUGS OR ALKALIES USED.

### THE HOSPITAL

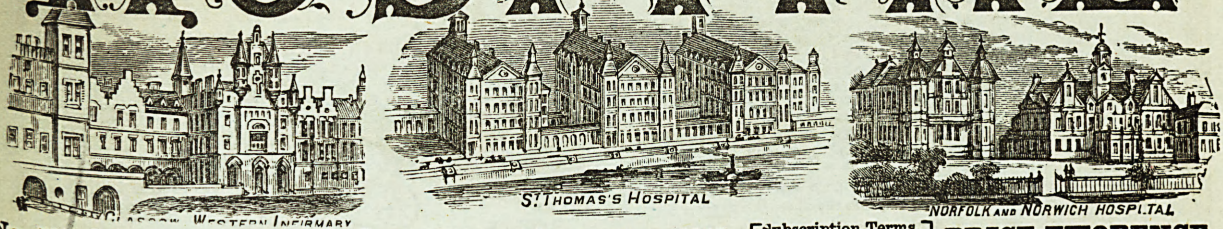

No. 599. Vol. XXIII. [Registered as a Newspaper.] SATURDAY, MARCH 19TH, 1898. [Subscription Terms, see p. 426] PRICE TWOPENCE.

